# Disorders of the Cholinergic System in COVID-19 Era—A Review of the Latest Research

**DOI:** 10.3390/ijms23020672

**Published:** 2022-01-08

**Authors:** Marta Kopańska, Marta Batoryna, Paulina Bartman, Jacek Szczygielski, Agnieszka Banaś-Ząbczyk

**Affiliations:** 1 Department of Pathophysiology, Institute of Medical Sciences, Medical College of Rzeszow University, 35-959 Rzeszow, Poland; 2 Sensusmed, Psychotherapy and Neurorehabilitation Center, 30-084 Cracow, Poland; m.batoryna@gmail.com; 3 Students Science Club “Reh-Tech”, University of Rzeszow, 35-959 Rzeszow, Poland; bartman.paulina@wp.pl; 4 Department of Neurosurgery, Institute of Medical Sciences, Medical College of Rzeszow University, 35-959 Rzeszow, Poland; jacek.szczygielski@vp.pl; 5 Department of Neurosurgery, Faculty of Medicine, Saarland University, 66424 Homburg, Germany; 6 Departament of Biology, Institute of Medical Sciences, Medical College of Rzeszow University, 35-959 Rzeszow, Poland; agnieszkabanas@o2.pl

**Keywords:** cholinergic system, COVID-19, SARS-CoV-2, acetylcholine, acetylcholinesterase, nicotinic receptors

## Abstract

The appearance of the SARS-CoV-2 virus initiated many studies on the effects of the virus on the human body. So far, its negative influence on the functioning of many morphological and physiological units, including the nervous system, has been demonstrated. Consequently, research has been conducted on the changes that SARS-CoV-2 may cause in the cholinergic system. The aim of this study is to review the latest research from the years 2020/2021 regarding disorders in the cholinergic system caused by the SARS-CoV-2 virus. As a result of the research, it was found that the presence of the COVID-19 virus disrupts the activity of the cholinergic system, for example, causing the development of myasthenia gravis or a change in acetylcholine activity. The SARS-CoV-2 spike protein has a sequence similar to neurotoxins, capable of binding nicotinic acetylcholine receptors (nAChR). This may be proof that SARS-CoV-2 can bind nAChR. Nicotine and caffeine have similar structures to antiviral drugs, capable of binding angiotensin-converting enzyme 2 (ACE 2) epitopes that are recognized by SARS-CoV-2, with the potential to inhibit the formation of the ACE 2/SARS-CoV-2 complex. The blocking is enhanced when nicotine and caffeine are used together with antiviral drugs. This is proof that nAChR agonists can be used along with antiviral drugs in COVID-19 therapy. As a result, it is possible to develop COVID-19 therapies that use these compounds to reduce cytokine production. Another promising therapy is non-invasive stimulation of the vagus nerve, which soothes the body’s cytokine storm. Research on the influence of COVID-19 on the cholinergic system is an area that should continue to be developed as there is a need for further research. It can be firmly stated that COVID-19 causes a dysregulation of the cholinergic system, which leads to a need for further research, because there are many promising therapies that will prevent the SARS-CoV-2 virus from binding to the nicotinic receptor. There is a need for further research, both in vitro and in vivo. It should be noted that in the functioning of the cholinergic system and its connection with the activity of the COVID-19 virus, there might be many promising dependencies and solutions.

## 1. Introduction

COVID-19 is a disease that is caused by the SARS-CoV-2 virus. The disease has many symptoms, ranging from asymptomatic, light conditions, where there is a cough, fever, headache, or loss of smell, to severe conditions requiring hospitalization [1].

The cholinergic (parasympathetic) system includes cholinergic structures, cholinergic receptors, and a neurotransmitter. The main centers of this system are located in the forebrain (basal part) and in the brainstem. Acetylcholine (ACh) is the most important neurotransmitter of the cholinergic system, produced and released by cholinergic neurons, and choline and coenzyme A are used for its synthesis. During the increased activity of the cholinergic system, choline is absorbed in greater amounts into the cytoplasm of neurons. ACh is stored in synaptic vesicles. The depolarization of the presynaptic membrane acts as an impulse to release acetylcholine into the synaptic cleft. Cholinergic neurons form among other neuromuscular synapses in the somatic system. ACh affects the neuromuscular plate and stimulates skeletal muscles to contract. It affects the muscarinic and nicotinic receptors and induces specific signal transduction pathways. Upon detachment from the receptor, acetylcholine is broken down by acetylcholinesterase (AChE) into choline and acetic acid. AChE inhibition causes an increased concentration of ACh in the synapse and triggers responses from both muscarinic and nicotinic receptors. Muscarinic receptors (mAChRs) and nicotinic receptors (nAChRs) are present in the cholinergic system. MAChRs are found in the cell membrane and regulate the function of ion channels. Under the influence of an extracellular transmitter, they initiate various processes. MAChRs are connected to the G protein, while nAChRs are ligand-gated membrane ionotropic receptors. Each receptor has at least two ligand binding sites located on the extracellular part of the receptor. The attachment of the ligand causes the opening/closing of the ion channel [2].

The immune system response is regulated by the autonomic nervous system. Inflammatory signals from the body travel to the brain via a cholinergic anti-inflammatory pathway, the main segment of which is the vagus nerve. Electrical stimulation (endo- or exogenous) of the vagus nerve leads to the increased release of Ach, which binds to nicotinic receptors on the macrophage cell membrane and induces the signal transduction pathway. This leads to a reduction in the synthesis of inflammatory cytokines and thus to inhibition of inflammation [2]. The cytokine storm is a pathological immune response associated with an excessive, dysregulated release of cytokines in response to infection. Its pathogenesis includes, inter alia, loss of control over the regulation of the production of pro-inflammatory cytokines (at the local and systemic level) [3]. During the coronavirus pandemic, the relationship between the severity of the course of the disease and poor condition of some patients and a cytokine storm was noticed. This evidence has triggered an increased interest in the search for new therapies in this field. The purpose of this article was to review the latest scientific reports from 2020/2021 on disorders in the cholinergic system caused by COVID-19. COVID-19 can also cause very similar diseases in children, such as Kawasaki disease (KD), COVID-19 related multi-system inflammatory syndrome (MIS-C), or COVID-19 hyperinflammation.

## 2. Literature Analysis

### 2.1. COVID-19 Can Cause Myasthenia Gravis 

**Figure 1:**
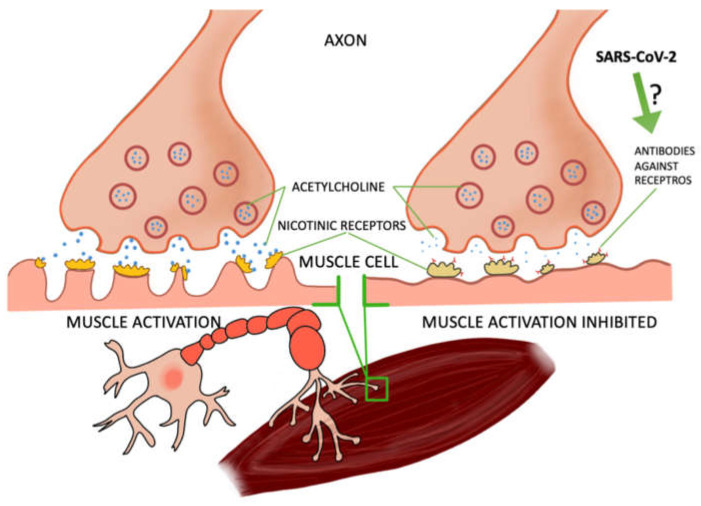
The relationship between the virus and the effect on muscle cells. Several reports from the real-world clinical practice have highlighted the noxious effects of SARS-CoV-2 on skeletal muscles.

Some of the studies were based on case reports of patients who contracted COVID-19 and developed myasthenia gravis, a disease of muscle weakness—particularly perilous if affecting the respiratory muscles. Myasthenia gravis is caused by the production of antibodies against nicotinic receptors in the neuromuscular junction, which reduces the number of functioning receptors by competitive blockade, increased receptor degeneration, or complement-mediated lysis of receptors [4,5]. In most of the patients, after the tests the presence of these antibodies was found [6,7,8,9,10] and one patient developed another type, anti-MusK, which blocks the formation of acetylcholine complexes in the synapses. In a study by Frida Essajee et al., experts from Stellenbosch University [8] described the recurrence of ocular myasthenia gravis caused by SARS-CoV-2 infection, resulting from the presence of ACE 2 receptors (angiotensin-converting enzyme 2), which are necessary for virus binding by cells. COVID-19 has a high affinity for these receptors, causing inflammation. In the research work of Domenico A. Restivo et al., three patients with myasthenia gravis were described. In a 64-year-old man, a sensory loss of 57% was noted after facial nerve stimulation, which is confirmation of the involvement of the postsynaptic neuromuscular junction. A postsynaptic deficit in neuromuscular transmission of the facial nerves (52%) and the ulnar nerves (21%) was also noted in a 68-year-old patient. However, in a 71-year-old patient, the result for COVID-19 was negative (in the previous two patients it was positive). The patient also had a postsynaptic deficit in the ulnar nerve (52%) [9]. M. Huber et al. described the case of a 21-year-old patient with myasthenia gravis symptoms, including double vision. About 4 weeks earlier, the woman had signs of mild COVID-19 infection (pain in the extremities and headaches accompanied by anosmia). Antibodies to SARS-CoV-2 were detected in the serum, and the edrophonium chloride test confirmed the diagnosis of myasthenia [10]. Moreover, several studies have concluded that the SARS-CoV-2 protein can, such as other viruses, cause latent myasthenia gravis in a patient. The molecular mimicry between SARS-CoV-2 and the nicotinic acetylcholine receptor may also be responsible for the development of this disease. It is not fully understood how exactly COVID-19 contributes to the development of myasthenia gravis. There is a need for further research in this area. It is also not precisely known how and whether SARS-CoV-2 may trigger myasthenia gravis in subjects who did not suffer from it before [6,7,8,11,12]. It also noted the increasing occurrence of COVID-19 related multi-system inflammatory syndrome (MIS-C) among children in Africa. Fifty two percent of the children participating in the study had to be hospitalized due to a dysfunction of the heart muscle [3]. In a study by Maxim Beydon et al., a case of a patient with secondary myositis due to COVID-19 was described. Symptoms such as muscle pain and muscle weakness appeared suddenly, and the patient did not show any disturbing signals concerning the upper or lower respiratory tract; tests for COVID-19 were negative and only on the 11th day was the presence of the SARS-CoV-2 virus found—the patient was still in a critical condition at the time [13] (Figure 1).

### 2.2. The Relationship between COVID-19 and Acetylcholinesterase Activity

An additional link between SARS-CoV-2 and the cholinergic system may be found while analyzing the activity of acetylcholinesterase, an enzyme responsible for the physiological breakdown of ACh in postsynaptic area. A. Courties et al. conducted a study on a group of 37 people with COVID-19, where only one patient was an active smoker with the probability of confounding effect of nicotine abuse on acetylcholinesterase activity. The control group consisted of 14 healthy people. The research results showed that the expression of CHRFAM7A (the duplicated isoform of the human α7 neuronal nicotinic acetylcholine receptor gene), which is one of the cholinergic genes, was reduced in patients with COVID-19; it also depended on the severity of the disease, which was much lower in patients with critical COVID-19 [14]. Attention should also be paid to the relationship between the lack of CHRFAM7A expression and increased activity of the TNF (tumor necrosis factor) signaling pathway, which was not observed in patients with a very severe course of COVID-19. The decreased expression of this gene in COVID-19 patients was associated, among others, with increased expression of genes in the TNF signaling pathway, which may suggest that during a cytokine storm, the reduction of the CHRFAM7A duplicate can be treated as a compensatory mechanism compensating for an inappropriate inflammatory response. Loss of relationships between positively correlated TNF levels with CHRFAM7A may lead to dysregulation of the cholinergic system during inflammation [14]. A. Courties et al. theorized whether the reduced level/absence of the CHRFAM7A gene could cause greater efficiency in the production of acetylcholine, which would mean that this mechanism is exceeded in patients with severe COVID-19, and that such patients may be more likely to succumb to a critically severe form of COVID-19. It was also noticed that younger patients had no or decreased expression of CHRFAM7A, which resulted in a worse course of the disease. This study encourages further discussion and leads to an intensified observation of the influence of the dominant negative CHRFAM7A double expression on the nicotinic response, which will allow for the future specification of the treatment scheme for manipulation of the cholinergic system. Additionally, they found that AChE was expressed by almost all controls and patients. The enzymatic activity of AChE might be important to evaluate before manipulating the cholinergic system in COVID-19 patients. They did not find any significant expression of the native CHRNA7 subunit, which was not surprising since the expression of the native CHRNA7 subunit is mostly present in the neuronal system and resident cells and much less in the non-neuronal system, especially in the circulating cells [15,16]. ChAT expression was not detectable in either whole blood RNA, which could be due to the method of detection since Kate Webb et al. analyzed whole blood expression and not specific expression after cell sorting. Particular attention was also drawn to the fact that the activity of acetylcholinesterase may have an impact on the cholinergic system of COVID-19 patients. The issue of Janus kinase (JAK) inhibition, which can affect inflammation but also viral entry into cells during COVID-19, has also been discussed. It is recommended that all severely ill patients should be screened for hyperinflammation in order to identify a subgroup of patients in whom immunosuppression may improve the reduction of mortality [17].

### 2.3. Interactions between Proteins

Recently, research conducted by many centers has also focused on the relationship between the binding of the SARS-CoV-2 virus with the nicotinic receptor (nAChR) and whether nicotine/caffeine can block this reaction [18,19,20,21]. Moreover, the observation of a small number of hospitalized smokers led to the hypothesis that nicotine may have a protective effect [22]. This issue has been addressed because the viral proteins have a similar sequence to nicotinic receptor antagonists; it turned out that the viral protein can affect the nAChR [18,19,20,21,23]. Furthermore, the penetration of the coronaviruses (including SARS-CoV-2) depends on the binding of the S proteins of the virus (spike proteins) to cell receptors and the stimulation by the S protein of the host cells. It is not clear what factors the virus uses to enter the cell, but this knowledge is crucial as it will allow us to better understand the transmission of the virus and to potentially find appropriate therapeutic solutions [16]. For this reason, the affinity of nicotine/caffeine for the ACE 2 receptor (at 6LZG, 6VW1 sites) was also studied, and the combination of nicotine/caffeine with antiviral drugs (as potential substances for association with ACE 2) was investigated. Thanks to the molecular dynamics simulations technique, the results were obtained, suggesting that this is a promising procedure as these factors can block the association of SARS-CoV-2 with the ACE 2 receptor [19,24,25]. According to Michaela Letko et al.’s experiment, the chimeric SARS-CoV-2 spike protein was incorporated into particles similarly to other clade 1 chimeric spikes and it was capable of entering the cells expressing human ACE 2, but not any of the other receptors tested [24]. The combinations with the highest affinity to ACE 2 are nicotine and favipiravir (6LZG—blocked) and a combination of caffeine and ribavirin (6VW1—blocked). This interaction is a possible reason for the epidemiologic fact that the number of current smokers hospitalized in connection with the SARS-CoV-2 epidemic in China is lower than expected compared to the prevalence of smoking in the country. A direct interaction between SARS-CoV-2 and the nicotinic receptor is possible, which can lead to inflammation in the course of COVID-19. As to the therapeutic potential, nicotinic receptor agonists can protect and restore nicotinic receptor function. Furthermore, there is evidence that nicotine and caffeine can bind to the SARS-CoV-2 S protein active sites as well as to ACE 2 receptors. ACE 2 expression is, i.a., race-dependent: thus, men in Asia may have higher tissue ACE 2 expression that is associated with estrogen levels, which is also involved in increasing ACE 2 expression and activity [26]. One of the unexpected findings was the similarity of the epitope on SARS-CoV glycoproteins and the SARS-CoV-2 S protein, which coincide with the sequence of the toxin that interacts with the nicotinic receptor; as a result, certain mutations may protect the epitope from binding to the nAChR. According to Changeux et al., the nicotinic acetylcholine receptor (nAChR) plays a key role in the pathophysiology of COVID-19. They proposed nicotine, nicotinic orthosteric, and/or allosteric agents as a possible therapy for SARS-CoV-2 infection [27].

It was also noticed that in the α7 complexes bound to the Y674–R685 peptide (the region corresponding to the S peptide of the virus), strong interactions in the aromatic box may occur, which raises a very important question: does it promote α7 nAChR gating [19]. This is an important issue that needs to be investigated, as activation of this subtype causes anti-inflammatory mechanisms in inflammatory cells, leading to a reduction in cytokine production. 

### 2.4. Vagus Nerve Stimulation—A Potential Therapy for COVID-19

On the topic of therapeutic strategies, one of them is the stimulation of the cholinergic system via, e.g., vagus nerve activation. Non-invasive vagus nerve stimulation is a neuromodulatory therapy that is U.S. Food and Drug Administration approved for acute and preventive treatment of migraines and cluster headaches. Vagus nerve stimulation may positively influence the condition of COVID-19 patients by suppressing inflammatory cytokine levels as a result of activation of the cholinergic inflammatory pathway [28]. Two patients diagnosed with COVID-19 participated in the study. One patient used 120-second stimulations on both sides of the neck approximately every 3 hours, with two additional stimulations once a day as needed. The second patient received one 120-second stimulation on the right side of the neck, 2 or 3 times a day. In both cases, there was an improvement in the wellbeing of patients; this therapy is currently becoming more and more attractive [28]. Vagus nerve stimulation (non-invasive) can reduce the patient’s need for ventilator support (blocks or alleviates cytokine storms). Currently, two methods can be distinguished that can help with respiratory failure; stimulation of the vagus nerve can inhibit airway narrowing (caused by inflammation) through the parasympathetic reflex. The second alternative is based on the action of the cholinergic anti-inflammatory pathway. Its stimulation causes the binding of acetylcholine with nicotinic receptors (α7nAChRs). As a result, it causes a reduced production of inflammatory cytokines in COVID-19 (mainly interleukin-6) [14,17,27,28]. That the vagus nerve was considered a potential controller of cytokine expression seemed likely because it is a primitive extension of the CNS into organs including the spleen and liver, which are the source of harmful cytokines. The vagus nerve also carries information to the brain, which is necessary to trigger fever and other responses to injury or infection and thus may serve as the sensory arm of the nerve loop’s inflammatory reflex that inhibits cytokine production [29].

## 3. Cytokine Storm and COVID-19

Cytokine production and action seem to play a pivotal role in the course of COVID-19, as SARS-CoV-2 infection causes the secretion of a large number of pro-inflammatory factors. The most important of all factors associated with the cytokine storm is IL-6 (interleukin-6), the level of which correlates strongly with the risk of death from COVID-19. Therefore, mitigating the cytokine storm may be an important approach, potentially reducing mortality in patients with this disease.

To identify the inflammatory response in the patient, the researchers assessed the levels of proteins associated with immune system activation in patients with mild, moderate, and severe disease, compared with a control group of healthy individuals. The results showed the dominant hyperinflammatory environment, as well as the relationship of biomarkers with the patient’s response measured on the ordinal scale in patients with COVID-19. Inhibition of Janus kinase proteins with baricitinib has also been shown to attenuate circulating biomarkers associated with cytokine storms. They indicate a relationship with the severity of the disease, and therefore may be helpful in selecting therapeutic options for patients with a different course of COVID-19 [30]. Among other drugs, candesartan, an angiotensin receptor antagonist, has demonstrated similar potential for action as it lowers the level of genes that are involved in the cytokine storm in COVID-19. The results of this study suggest that angiotensin receptor blockers (ARB treatment) may be beneficial in treating the severe course of COVID-19 by alleviating increased inflammation. Thus, the anti-inflammatory properties of candesartan suppress the cytokine storm associated with this disease [31]. Following the concept of ACE system participation in COVID-19 pathophysiology, Zhen Wang et al. developed an inhaled modular microfluidic microsphere aerosol that neutralizes viruses and cytokines in COVID-19. In the production of this experimental drug, a genetically modified membrane made of ACE 2 receptor cells and pro-inflammatory macrophages (highly expressive cells) was used. This aerosol was effective in the entire respiratory system, including the nasopharynx, trachea, and alveoli. The inhaled aerosol competes with SARS-CoV-2 by preventing it from attacking targeted cells throughout the airways. This aerosol also alleviates the cytokine storm [32]. Another pathway that has been taken into account as participating in the COVID-19 course was based on haem oxygenase-1 (HO-1) function. This is a stress-induced enzyme that exhibits cytoprotective, anti-apoptotic, and immunomodulatory properties through its by-products, which may have an influence on the modulation of inflammation related conditions. HO-1 may be a potential therapy for COVID-19 as it is able to moderate the cytokine storm. It may also be an interesting target for controlling COVID-19 by inhibiting SARS-CoV-2 replication. In sum, targeting the HO-1 function provides an opportunity to both control infection with this virus and to reduce cytokine storm along with acute respiratory distress syndrome (ARDS) post COVID-19 [33]. It has also been hypothesized that phenyl metamizole (C10) may be a potential drug in all diseases that are based on a cytokine storm. Preclinical and clinical studies are still needed before this drug is available, however animal studies have shown no relevant toxic effects of this compound. There was also no anti-thyroid effect (being the initial concern as to the side effects of the mentioned drug) [33,34]. Further, it has also been noted that cyclooxygenase (COX) inhibitors have the potential to inhibit the cytokine storm (since etoricoxib can inhibit pro-inflammatory factors). COX, being one of the key executors of inflammatory reaction, has two isoenzymes: COX-1 and COX-2; the latter manifested in pathological conditions, and its inhibitor is etoricoxib. This compound has some side effects, but these are usually mild and well tolerated. Etoricoxib may be beneficial for intestinal and renal symptoms in patients with COVID-19 because the use of the selective COX-2 inhibitors has a relatively low risk of damage to the lining of the stomach or renal hemodynamics [34]. However, a thorough evaluation of etoricoxib should be performed before it can be securely repurposed as a treatment for COVID-19. Another therapy concept, which is linked to the inflammatory response, is based on intravenous therapy with a high dose of vitamin C (24 g/day). Vitamin C plays an important role in immunomodulation; it can inhibit the activation of the nuclear factor kappa-B, the main pro-inflammatory transcription factor, and is also important in the overall immunity of the body. Vitamin C may also inhibit the production of IL-6 and may also act as a cytokine regulator [35].

## 4. Pediatric Multisystem Inflammatory Syndrome, Kawa-COVID-19, and Hyperinflammatory Syndrome in COVID-19

Another specific condition related to inflammatory activation and thus possibly linked to SARS-CoV-2 infection is Kawasaki disease (KD). There has been an increase in the number of patients with COVID-19 overlapping with KD, also known as the pediatric multisystem inflammatory syndrome (MIS-C). KD and MIS-C are similar in that they show several common symptoms such as skin rash, enlarged lymph nodes, strawberry tongue, and elevated inflammatory biomarkers. However, MIS-C in COVID-19 also has its own unique features, e.g., the disease occurs even in older children and adolescents, and the presence of specific abdominal symptoms as well as left ventricular systolic dysfunctions [36]. KD (related to COVID-19), on the other hand, is defined by a fever that lasts five days and is associated with at least four other symptoms: conjunctivitis, lymphadenopathy, skin rash, red and chapped lips, and inflammation of the hands and feet. The whole clinical picture of this condition is usually rounded up by elevation of inflammation markers, e.g., ESR, CRP, or procalcitonin. MIS-C is also found on the basis of laboratory evidence of inflammation, i.e., increased inflammatory markers, fibrinogen, D-dimer, ferritin, LDH, IL-6, and the presence of neutrophilia or lymphopenia [37]. MIS-C can occur in people up to the age of 21. In a study conducted in France, patients who met KD criteria were selected for comparison with the pre-COVID-19 pandemic group. Twelve patients reported contacting individuals with confirmed COVID-19 or a high suspicion of COVID-19. COVID-19 infection was confirmed in twelve patients. Ten patients met the American Heart Association (AHA) definition of total KD. Fever above 39 ° C occurred in all patients (16), and above 40 ° C in seven patients. The main symptoms were mucocutaneous involvement, skin rash, rash/swelling of the feet and hands, conjunctivitis, dry and chapped lips, cervical lymphadenopathy, and arthritis. Less common symptoms included gastrointestinal symptoms and hemodynamic failure, tachycardia and one of the following symptoms: arterial hypotension, cold extremities, decreased peripheral pulse, and orchitis. There were also less common neurological symptoms that could be related to COVID-19, e.g., headaches, aseptic meningitis, respiratory symptoms (cough and shortness of breath), Raynaud’s syndrome, and anosmia. However, no pulmonary embolism or acute respiratory distress syndrome were observed. Finally, patients with Kawasaki disease and COVID-19 (Kawa-COVID-19) were compared to the group studied prior to the COVID-19 pandemic. The main differences noted were the presence of KD in elderly patients, a lower number of platelets, and lymphocytes. In people with Kawa-COVID-19, a higher incidence of myocarditis and pericarditis was observed [38,39,40]. The frequency of Kawa-COVID-19 symptoms are presented in the Table 1. Suggestions were made that Kawa-COVID-19 (an autoinflammatory disease similar to KD) is associated with SARS-CoV-2 infection. Assessment of host immunological and genetic factors is essential for a better understanding of this new disease entity [38,39]. There are also reports of multi-system inflammation in children that mimics Kawasaki disease, or pediatric multisystem inflammatory syndrome correlated with COVID-19 (MIS-C). The definition of this disease has been developed by the Royal College of Paediatrics and Child Health, Centers for Disease Control and Prevention, and the WHO. To diagnose MIS-C, the following conditions must be met: a person under the age of 21 with persistent fever, inflammation, and symptoms of dysfunction of one or more organs, shock, disturbances in heart rate and respiratory system, kidneys, gastrointestinal tract or neurological disorders, other microbiological causes (e.g., bacterial sepsis) are excluded, a positive COVID-19 test, recent SARS-CoV-2 infection, or exposure to COVID-19 in the last 4 weeks prior to the onset of MIS-C symptoms [40]. 

Children with COVID-19 may develop a hyperinflammatory reaction similar to that seen in adults. Due to the fact that MIS-C has many definitions, it was decided that a special research team would be appointed, whose work will provide guidance on the assessment and treatment of MIS-C and COVID-19-related hyperinflammatory syndromes in children. During both the first and second considerations, the statements that children taking moderate and high doses of immunosuppressants are more likely to develop severe COVID-19 remained unchanged. Both adults and children have similar symptoms of COVID-19 (fever, abdominal pain, diarrhea, and respiratory symptoms). Children who develop respiratory symptoms due to COVID-19 should receive immunomodulatory therapy in which the use of glucocorticosteroids may be considered. Treatment with anakinra appears to be safe in severe infections and hyperinflammatory syndromes in children. Therefore, it is worth considering this form of therapy in patients with disease resistant to treatment despite the use of glucocorticosteroids, as well as in patients with contraindications to steroid therapy. It has been concluded by subsequent considerations from the designated study group that tocilizumab should not be used in children and adolescents with COVID-19 associated with hyperinflammation as no benefit was noted in randomized, double-blind studies in adults with COVID-19 pneumonia. In addition, the effects of this substance are long lasting, which can cause problems if the patient reacts badly to the medicine. It is also noted that there is insufficient evidence to support the use of other immunomodulatory drugs, unless the use of Il-1 blocking therapies or the use of glucocorticosteroids has completely failed and did not bring the expected results [41].

## 5. Conclusions

The impact of COVID-19 on the cholinergic system is an area that, due to multiplicity of possible merging points of pathophysiological processes, should be studied more intensively. So far, several studies have shown that COVID-19 causes a clear dysregulation of the cholinergic system. This observation will guide further research, as there are many promising therapies that will prevent the SARS-CoV-2 virus from binding to the nicotinic receptor. Nicotine agonists (e.g., nicotine/caffeine) can bind to the nAChR, thereby preventing inflammation by activating this receptor. A similar effect is expected of vagus nerve stimulation, which is a potential therapy for COVID-19. Thus, the action of the cholinergic system bears great therapeutic potential for COVID-19 cases, which needs to be investigated further, both in vitro and in vivo. The knowledge about SARS-CoV-2-related syndromes in children is relatively low compared to the adult form of the disease, probably due to the epidemiologic profile of SARS-CoV-2 infection, with less severe manifestation in the pediatric group. Nevertheless, several particular conditions related to COVID-19 course in children, such as Kawa-COVID-19 or MIS-C, have already been identified. Selection of the appropriate and effective treatment strategies relies on a better understanding of COVID-19 pathophysiological background, with the cholinergic system being a recurrent theme in the growing body of scientific evidence.

## Figures and Tables

**Table 1:** The frequency of Kawa-COVID-19 symptoms.

Typical Symptoms for KD	Number of Patients	Less Common Symptoms	Number of Patients	Less Common Neurological Symptoms	Number of Patients
Fever over 39 °C	16	Gastrointestinal symptoms	13	Headache	6
Fever over 40 °C	7	Hemodynamic failure	11	Aseptic meningitis	3
Mucocutaneous involvement	15	Inflammation of the testicles	2	Respiratory symptoms	2
Skin rash	13	-	-	Raynaud Syndrome	2
Rash/swelling of the feet and hands	11	-	-	Retained	1
Conjunctivitis	15	-	-	-	-
Dry and chapped lips	14	-	-	-	-
Cervical lymphadenopathy	6	-	-	-	-
Arthritis	1	-	-	-	-

## References

[1] Fudim M., Qadri Y.J., Ghadimi K., MacLeod D.B., Molinger J., Piccini J.P., Whittle J., Wischmeyer P.E., Patel M.R., Ulloa L. (2020). Implications for neuromodulation therapy to control inflammation and related organ dysfunction in COVID-19. J. Cardiovasc. Transl. Res..

[2] Kruk-Słomka M., Budzyńska B., Biała G. (2012). Involvement of cholinergic receptors in the different stages of memory measured in the modified elevated plus maze test in mice. Pharmacol Rep.

[3] Bonaz B., Sinniger V., Pellissier S. (2020). Targeting the cholinergic anti-inflammatory pathway with vagus nerve stimulation in patients with COVID-19. Bioelectron. Med..

[4] Hübers A., Lascano A.M., Lalive P.H. (2020). Management of patients with generalised myasthenia gravis and COVID-19: Four case reports. J. Neurol. Neurosurg. Psychiatry.

[5] Meriggioli M.N., Sanders D.B. (2009). Autoimmune myasthenia gravis: Emerging clinical and biological heterogeneity. Lancet Neurol..

[6] Karimi N., Okhovat A.A., Ziaadini B., Ashtiani B.H., Nafissi S., Fatehi F. (2021). Myasthenia gravis associated with novel coronavirus 2019 infection: A report of three cases. Clin. Neurol. Neurosurg..

[7] Sriwastava S., Tandon M., Kataria S., Daimee M., Sultan S. (2021). New onset of ocular myasthenia gravis in a patient with COVID-19: A novel case report and literature review. J. Neurol..

[8] Essajee F., Lishman J., Solomons R., Abraham D.R., Goussard P., van Toorn R. (2021). Transient acetylcholine receptor-related myasthenia gravis, post multisystem inflammatory syndrome in children (MIS-C) temporally associated with COVID-19 infection. BMJ Case Rep..

[9] Beydon M., Chevalier K., al Tabaa O., Hamroun S., Delettre A., Thomas M., Herrou J., Riviere E., Mariette X. (2020). Myositis as a manifestation of SARS-CoV-2. Ann. Rheum. Dis..

[10] Restivo D.A., Centonze D., Alesina A., Marchese-Ragona R. (2020). Myasthenia gravis associated with SARS-CoV-2 infection. Ann. Int. Med..

[11] Assini A., Gandoglia I., Damato V., Rikani K., Evoli A., del Sette M. (2021). Myasthenia gravis associated with anti-MuSK antibodies developed after SARS-CoV-2 infection. Eur. J. Neurol..

[12] Dunsire M.F., Clarke S.G., Stedmon J.J. (2001). Undiagnosed myasthenia gravis unmasked by neuromuscular blockade. Br. J. Anaesth..

[13] Webb K., Abraham D., Faleye A., McCulloch M., Rabie H., Scott C. (2020). Cape town MISC-team, multisystem inflammatory syndrome in children in South Africa. Lancet Child Adolesc. Health.

[14] Courties A., Boussier J., Hadjadj J., Yatim N., Barnabei L., Péré H., Veyer D., Kernéis S., Carlier N., Pène F. (2021). Regulation of the acetylcholine/α7nAChR anti-inflammatory pathway in COVID-19 patients. Sci. Rep..

[15] Tracey K.J. (2007). Physiology and immunology of the cholinergic antiinflammatory pathway. J. Clin. Investig..

[16] Gonzalez-Rubio J., Navarro-Lopez C., Lopez-Najera E., Lopez-Najera A., Jimenez-Diaz L., Navarro-Lopez J.D., Najera A. (2020). Cytokine release syndrome (CRS) and nicotine in COVID-19 patients: Trying to calm the storm. Front. Immunol..

[17] Ciaglia E., Vecchione C., Puca A.A. (2020). COVID-19 infection and circulating ACE2 levels: Protective role in women and children. Front. Pediatrics.

[18] Mohammadi S., Heidarizadeh M., Entesari M., Esmailpour A., Esmailpour M., Moradi R., Sakhaee N., Doustkhah E. (2020). In silico investigation on the inhibiting role of nicotine/caffeine by blocking the S protein of SARS-CoV-2 versus ACE2 receptor. Microorganisms.

[19] Oliveira A.S.F., Ibarra A.A., Bermudez I., Casalino L., Gaieb Z., Shoemark D.K., Gallagher T., Sessions R.B., Amaro R.E., Mulholland A.J. (2021). A potential interaction between the SARS-CoV-2 spike protein and nicotinic acetylcholine receptors. Biophys. J..

[20] Farsalinos K., Eliopoulos E., Leonidas D.D., Papadopoulos G.E., Tzartos S., Poulas K. (2020). Nicotinic cholinergic system and COVID-19: In silico identification of an interaction between SARS-CoV-2 and nicotinic receptors with potential therapeutic targeting implications. Int. J. Mol. Sci..

[21] Lagoumintzis G., Chasapis C.T., Alexandris N., Kouretas D., Tzartos S., Eliopoulos E. (2021). Konstantinos farsalinos, konstantinos poulas, nicotinic cholinergic system and COVID-19: In silico identification of interactions between α7 nicotinic acetylcholine receptor and the cryptic epitopes of SARS-Co-V and SARS-CoV-2 spike glycoproteins. Food Chem. Toxicol..

[22] Smith M., Smith J.C. (2020). Repurposing therapeutics for COVID-19: Supercomputer-based docking to the SARS-CoV-2 viral spike protein and viral spike protein-human ACE2 interface. ChemRxiv.

[23] Farsalinosab K., Niaura R., le Houezec J., Barbouni A., Tsatsakis A., Kouretas D., Vantarakis A., Poulas K. (2020). Editorial: Nicotine and SARS-CoV-2: COVID-19 may be a disease of the nicotinic cholinergic system. Toxicol. Rep..

[24] Hoffmann M., Kleine-Weber H., Schroeder S., Krüger N., Herrler T., Erichsen S., Schiergens T.S., Herrler G., Wu N.-H., Nitsche A. (2020). SARS-CoV-2 cell entry depends on ACE2 and TMPRSS2 and is blocked by a clinically proven protease inhibitor. Cell.

[25] Letko M., Marzi A., Munster V. (2020). Functional assessment of cell entry and receptor usage for SARS-CoV-2 and other lineage B betacoronaviruses. Nat. Microbiol..

[26] Tizabi Y., Getachew B., Copeland R.L., Aschner M. (2020). Nicotine and the nicotinic cholinergic system in COVID-19. FEBS J..

[27] Changeux J.P., Amoura Z., Rey F.A., Miyara M. (2020). A nicotinic hypothesis for COVID-19 with preventive and therapeutic implications. C. R. Biol..

[28] Mehta P., McAuley D.F., Brown M., Sanchez E., Tattersall R.S., Manson J.J. (2020). HLH across speciality collaboration, COVID-19: Consider cytokine storm syndromes and immunosuppression. UK Lancet.

[29] Staats P., Giannakopoulos G., Blake J., Liebler E., Levy R.M. (2020). The use of non-invasive vagus nerve stimulation to treat respiratory symptoms associated with COVID-19: A theoretical hypothesis and early clinical experience. Neuromodul. Technol. Neural Interface.

[30] Ye Q., Wang B., Mao J. (2020). The pathogenesis and treatment of the ‘Cytokine Storm’ in COVID-19. J. Infect..

[31] Sims J.T., Krishnan V., Chang C.-Y., Engle S.M., Casalini G., Rodgers G.H., Bivi N., Nickoloff B.J., Konrad R.J., de Bono S. (2020). Characterization of the cytokine storm reflects hyperinflammatory endothelial dysfunction in COVID-19. J. Allergy Clin. Immunol..

[32] Elkahloun A.G., Saavedra J.M. (2020). Candesartan could ameliorate the COVID-19 cytokine storm. Biomed. Pharmacother..

[33] Wang Z., Xiang L., Lin F., Cai Z., Ruan H., Wang J., Liang J., Wang F., Lu M., Cui W. (2021). 456 inhaled ACE2-engineered microfluidic microsphere for intatacheal neutralization of COVID-19 and calming of the cytokine 457 storm. Matter.

[34] Rossi M., Piagnerelli M., van Meerhaeghe A., Bodjeltia K.Z. (2020). Heme oxygenase-1 (HO-1) cytoprotective pathway: A potential treatment strategy against coronavirus disease 2019 (COVID-19)-induced cytokine storm syndrome. Med. Hypotheses.

[35] Giuliani C., Bucci I., Napolitano G. (2021). Phenylomethimazole is a candidate drug for treatment of serve forms of coronavirus disease 2019 (COVID-19) as well as other virus-induced “cytokine storm”. Med. Hypotheses.

[36] Wang R. (2021). Etoricoxib may inhibit cytokine storm to treat COVID-19. Med. Hypotheses.

[37] Liu F., Zhu Y., Zhang J., Li Y., Peng Z. (2020). Intravenous high-dose vitamin C for the treatment of serve COVID-19: Study protocol for a multicentre randomized controlled trial. BMJ Open.

[38] Ebina-Shibuya R., Namkoong H., Shibuya Y., Horita N. (2020). Multisystem inflammatory syndrome in children (MIS-C) with COVID-19: Insights from simultaneous familial Kawasaki Disease cases. Int. J. Infect. Dis..

[39] Pouletty M., Borocco C., Ouldali N., Caseris M., Basmaci R., Lachaume N., Bensaid P., Pichard S., Kouider H., Morelle G. (2020). Pediatric multisystem inflammatory syndrome temporally associated with SARS-CoV-2 mimicking Kawasaki disease (Kawa-COVID-19): A multicentre cohort. Ann. Rheum. Dis..

[40] Simpson J.M., Newburger J.W. (2020). Multisystem inflammatory syndrome in children in association with COVID-19. Circulation.

[41] Henderson L.A., Canna S.W., Friedman K.G., Gorelik M., Lapidus S.K., Bassiri H., Behrens E.M., Ferris A., Kernan K.F., Schulert G.S. (2021). American college of rheumatology clinical guidance for multisystem inflammatory syndrome in childern associated with SARS-CoV-2 and hyperinflammatrion in pediatric COVID-19. Arthritis Rheumatol..

